# A gonadotropin-releasing hormone antagonist reduces serum adrenal androgen levels in prostate cancer patients

**DOI:** 10.1186/s12894-017-0261-z

**Published:** 2017-08-29

**Authors:** Yoshiyuki Miyazawa, Yoshitaka Sekine, Takahiro Syuto, Masashi Nomura, Hidekazu Koike, Hiroshi Matsui, Yasuhiro Shibata, Kazuto Ito, Kazuhiro Suzuki

**Affiliations:** Department of Urology, Gunma University Graduate School of Medicine, 3-9-22 Showa-machi, Maebashi, Gunma 371-8511 Japan

**Keywords:** Prostate cancer, GnRH antagonist, Adrenal androgen

## Abstract

**Background:**

Adrenal androgens play an important role in the development of castration-resistant prostate cancer therapeutics. The effect of gonadotropin-releasing hormone (GnRH) antagonists on adrenal androgens has not been studied sufficiently. We measured testicular and adrenal androgen levels in patients treated with a GnRH antagonist.

**Methods:**

This study included 47 patients with histologically proven prostate cancer. All of the patients were treated with the GnRH antagonist degarelix. The mean patient age was 73.6 years. Pre-treatment blood samples were collected from all of the patients, and post-treatment samples were taken at 1, 3, 6, and 12 months after starting treatment. Testosterone (T), dihydrotestosterone (DHT), dehydroepiandrosterone (DHEA), 17β-estradiol (E2), and androstenedione (A-dione) were measured by liquid chromatography-mass spectrometry. Dehydroepiandrosterone-sulfate (DHEA-S), luteinizing hormone, and follicle-stimulating hormone levels were measured by electro-chemiluminescence immunoassays.

**Results:**

A significant reduction in T level (97.3% reduction) was observed in the patients 1 month after initiating treatment. In addition, levels of DHT, E2, DHEA-S, and A-dione decreased 1 month after initiating treatment (93.3, 84.9, 16.8, and 35.9% reduction, respectively). T, DHT, E2, DHEA-S, and A-dione levels remained significantly suppressed (97.1, 94.6, 85.3, 23.9, and 40.5% reduction, respectively) 12 months after initiating treatment. A significant decrease in DHEA level (15.4% reduction) was observed 12 months after initiating treatment.

**Conclusions:**

Serum adrenal androgen levels decreased significantly in patients treated with a GnRH antagonist. Thus, long-term GnRH antagonist treatment may reduce serum adrenal androgen levels.

## Background

Prostate cancer has become one of the most prevalent diseases among men in Western countries, and its incidence has increased in Japan [1]. The disease is dependent on androgens; therefore, patients are often treated with androgen deprivation therapy [2]. Gonadotropin-releasing hormone (GnRH) antagonists block receptors directly, thereby rapidly suppressing testosterone (T) without the T surge and flare. No clinical studies on the GnRH antagonist degarelix have reported evidence of a T surge or flare [3–6]. Various drugs that interact with androgen receptors (ARs) have been developed and used clinically. In addition to “conventional” AR inhibitors such as flutamide [7] and bicalutamide [8], enzalutamide prolongs survival time before and after chemotherapy [9, 10]. Abiraterone, an androgen biosynthetic enzyme inhibitor, also improves patient prognosis [11]. Adrenal androgens are important hormones for developing therapy for castration-resistant prostate cancer. Although circulating adrenal androgens are mainly secreted from the adrenal glands, the testes secrete about 10% of the total [12]. In addition, the role of aberrant luteinizing hormone (LH) receptor expression in the adrenal glands has been studied in patients with adrenocorticotropic hormone (ACTH)-independent Cushing’s syndrome [13–15]. We previously reported that long-term luteinizing hormone-releasing hormone (LH-RH) agonist treatment reduced adrenal androgen levels via LH receptors in the adrenal cortex [16]. However, the effects of GnRH antagonists on adrenal androgens have not been studied sufficiently. In this study, we evaluated the effects of a GnRH antagonist on changes in serum adrenal androgen levels.

## Methods

### Patients

We studied 47 patients diagnosed with prostate cancer pathologically at Gunma University Hospital (Maebashi, Japan). Table 1 shows the clinical characteristics of the enrolled patients, who ranged in age from 60 to 87 years (mean, 73.6 years). The clinical stages of the patients were T1cN0M0 (*n* = 8), T2N0M0 (*n* = 14), T3N0M0 (*n* = 9), T4N0M0 (*n* = 2), TanyN1M0 (*n* = 4), TanyN0M1 (*n* = 5), and TanyN1M1 (*n* = 5). Ten patients had distant metastases; seven had a bone metastasis and three had a visceral metastasis. All patients were administered degarelix as a monthly subcutaneous injection (240 mg for the first month followed by 12 maintenance doses of 80 mg). Four patients were treated with bicalutamide as a combined antiandrogen blockade. Of the 33 TanyN0M0 patients, 12 (mean age, 69.3; range, 60–77 years) underwent curative radiation therapy in combination with hormone therapy: 7 patients received intensity-modulated radiation therapy, 4 patients received carbon-ion radiotherapy, and 1 patient received high-dose brachytherapy. The other 21 patients of the 33 TanyN0M0 patients received hormone therapy either because of their age or their wishes (mean age, 76.8; range, 62–84 years). All 14 patients who had lymph node metastasis or distant metastasis (TanyN1M0: *n* = 4, TanyN0M1: *n* = 5, and TanyN1M1: *n* = 5) were treated with hormonal therapy. The Ethical Committee for Clinical Study of Gunma University Graduate School of Medicine approved this study, and written consent was obtained from all of the enrolled patients. We registered this clinical trial with the University Hospital Medical Information Network (UMIN ID: UMIN000011990).

**Table 1:** Clinical characteristics of the patients

*Characteristic*		
No. of patients	47	
Age (year, mean ± SD)	73.6 ± 7.02	
Initial PSA (median ± SD)	11.1 ± 489.1 ng/ml	
*Stage*	No. of patients	
T1cN0M0	8	17%
T2N0M0	14	29.8%
T3N0M0	9	19.1%
T4N0M0	2	4.3%
TanyN1M0	4	8.5%
TanyN0M1	5	10.6%
TanyN1M1	5	10.6%
*Metastasis*		
All distant metastasis	10	21.2%
Bone metastasis	7	14.9%
Visceral metastasis	3	6.4%
*Gleason Score*		
GS 6	4	9%
GS 7	15	32%
GS≧8	28	59%

*Abbreviations*: *PSA* Prostate Specific Antigen

### Blood samples and measurement of hormone levels

Pre-treatment blood samples were collected from all of the patients, and post-treatment samples were taken at 1, 3, 6, and 12 months after starting the treatment. All serum samples were stored at −80 °C prior to testing. T, dihydrotestosterone (DHT), 17β-estradiol (E2), dehydroepiandrosterone (DHEA), and androstenedione (A-dione) were measured by liquid chromatography-mass spectrometry. Dehydroepiandrosterone-sulfate (DHEA-S), LH, and follicle-stimulating hormone (FSH) were measured by electro-chemiluminescence immunoassays. To investigate the rapid T-lowering effect of degarelix, additional tests were conducted using serum 1 (*n* = 39) and 2 (*n* = 36) weeks after degarelix administration by electro-chemiluminescence immunoassays.

### Statistical analysis

All values are expressed as the mean ± standard deviation and were compared using Student’s *t*-test. A *p-*value <0.05 was considered significant. We used an analysis of variance (ANOVA) and the Tukey-Kramer method to analyze the changes in LH and FSH levels between the nadir and other time points.

## Results

Table 2 shows the changes in hormone levels during treatment. T levels decreased significantly (97.3% reduction) in GnRH antagonist-treated patients 1 month after initiating treatment compared to those at baseline. In addition, the lower T level was maintained until 12 months after initiating treatment (97.1% reduction). DHT and E_2_ decreased 1 month after initiating treatment (DHT, 93.2%; E_2_, 84.9% reduction, respectively) and these levels were maintained until 12 months after initiating treatment. DHEA-S and A-dione levels decreased significantly 1, 3, 6, and 12 months after initiating treatment and remained low until 12 months after the start of treatment (DHEA-S, 23.9%; A-dione, 40.5% reduction, respectively). We did not observe a decrease in DHEA levels 1, 3, or 6 months after initiating treatment, but the DHEA level was significantly lower 12 months after treatment compared to baseline (15.4% reduction). LH and FSH levels decreased 1 month after initiating treatment compared to baseline (LH, 96.0%; FSH, 93.9% reduction, respectively), and they reached the nadir 1 month after initiating treatment. An ANOVA detected no change between the nadir LH level and levels measured 3, 6, and 12 months after initiating treatment (*p* = 0.065). FSH levels increased gradually from 1 to 12 months after initiating treatment. We found a significant change between the FSH nadir level and the level 12 months after initiating treatment (*p* = 0.0006). In addition, we found a significant change between the levels 3 and 12 months after initiating treatment. The changes and statistical results for the nadir LH and FSH levels and the levels at the other time points are shown in Table 3. Examining the early decline in T using an electro-chemiluminescence immunoassay, the mean T level was 19.4 ± 9.11 ng/mL at 1 week after treatment (*n* = 39) and 11.8 ± 7.13 ng/mL at 2 weeks after (*n* = 36) treatment (Data is shown in Table 2).

**Table 2:** Changes of hormone levels in prostate cancer patients treated GnRH antagonist

	Pre	1 w	2 w	1 mo	3 mo	6 mo	12 mo	Statistics
T								*p* < 0.05
Measurement, ng/dL percentile change	376.66 ± 161.71	19.4 ± 9.11^†^ −94.8% (*n* = 39)	11.8 ± 7.13^†^ −96.7% (*n* = 36)	10.23 ± 5.09 −97.3%	10.09 ± 3.98 −97.3%	10.49 ± 4.52 −97.2%	10.81 ± 5.36 −97.1%	Pre vs 1 w, 2 w, 1 mo, 3 mo, 6 mo, 12 mo
DHT								*p* < 0.05
Measurement, pg/mL percentile change	442.76 ± 256.68			29.95 ± 15.89 −93.2%	27.09 ± 15.28 −93.9%	25.30 ± 14.48 −94.3%	24.12 ± 14.01 −94.6%	Pre vs 1 mo, 3 mo, 6 mo, 12 mo
E_2_								*p* < 0.05
Measurement, pg/mL percentile change	23.58 ± 12.81			3.56 ± 2.58 −84.9%	3.25 ± 2.21 −86.2%	3.32 ± 2.01 −85.9%	3.46 ± 2.37 −85.3%	Pre vs 1 mo, 3 mo, 6 mo, 12 mo
DHEA-S								*p* < 0.05
Measurement, μg/dL percentile change	125.47 ± 62.48			104.38 ± 56.03 −16.8%	103.7 ± 52.52 −17.3%	104.17 ± 57.03 −17.0%	95.47 ± 58.23 −23.9%	Pre vs 1 mo, 3 mo, 6 mo, 12 mo
DHEA								*p* < 0.05
Measurement, ng/mL percentile change	2.50 ± 1.46			2.19 ± 1.52 −12.0%	2.27 ± 1.45 −8.7%	2.23 ± 1.57 −10.8%	2.11 ± 1.69 −15.4%	Pre vs 12 mo
A-dione								*p* < 0.05
Measurement, ng/mL percentile change	0.715 ± 0.348			0.458 ± 0.278 −35.5%	0.472 ± 0.231 −33.9%	0.448 ± 0.230 −37.4%	0.426 ± 0.220 −40.5%	Pre vs 1 mo, 3 mo, 6 mo, 12 mo
LH								*p* < 0.05
Measurement, mIU/mL percentile change	7.20 ± 7.31			0.285 ± 0.385 −96.0%	0.255 ± 0.306 −96.5%	0.303 ± 0.355 −95.8%	0.453 ± 0.479 −93.7%	Pre vs 1 mo, 3 mo, 6 mo, 12 mo
FSH								*p* < 0.05
Measurement, mIU/mL percentile change	14.56 ± 14.47			0.889 ± 1.100 −93.9%	0.993 ± 0.100 −93.2%	1.224 ± 1.135 −91.6%	1.83 ± 1.424 −87.4%	Pre vs 1 mo, 3 mo, 6 mo, 12 mo

*Abbreviations*: *T*, Testosterone, *DHT* dihydrotestosterone, *E*_*2*_ Estradiol, *DHEA* dehydroepiandrosterone, *DHEA-S* Dehydroepiandrosterone-sulfate, *A-dione* androstenedione, *LH* Luteinizing hormone, *FSH* Follicle Stimulating Hormone, *Pre* pretreatment, *1,2 w* 1,2 weeks after initiation of GnRH antagonist treatment, 1,3,6,12 mo, 1,3,6,12 months after initiation of GnRH antagonist treatment

Percentile change indicates changes in comparison with pretreatment levels. Values are expressed as mean ± SD

†: Data of testosterone after 1 week (*n* = 39) and 2 weeks (*n* = 36) were measured by the ECLIA, and all other data were measured by LC-MS/MS

**Table 3:** The changes and statistical processing in LH and FSH levels of nadir and other points

	1 mo (nadir)	3 mo	6 mo	12 mo	Statistics
LH					
Measurement, mIU/mL	0.285 ± 0.385	0.255 ± 0.306	0.303 ± 0.355	0.453 ± 0.479	There were no significant changes between nadir and other points. ANOVA; *p* = 0.065
FSH					
Measurement, mIU/mL	0.889 ± 1.100	0.993 ± 0.100	1.224 ± 1.135	1.83 ± 1.424	There were significant changes between nadir and 12 mo, 3 mo and 12 mo.Tukey-Kramer methods; *p* = 0.0006

*Abbreviations*: *LH* Luteinizing hormone, *FSH* Follicle Stimulating Hormone. Values are expressed as mean ± SD

## Discussion

The serum adrenal androgen levels decreased significantly after treatment with a GnRH antagonist. To our knowledge, this is the first report to show a significant decrease in adrenal androgen levels in patients with prostate cancer treated long-term with a GnRH antagonist. Some studies have shown a relationship between LH-RH agonist therapy and changes in adrenal androgen levels. The DHEA-S levels decreased slightly albeit not significantly in patients with prostate cancer treated with a LH-RH agonist for 28 days [17]. Eri et al. [18] showed that the A-dione and DHEA-S levels of patients treated with a LH-RH agonist for 6 months for benign prostate hyperplasia decreased by 48 and 24%, respectively. Those authors showed that reduced testicular secretion of both hormones contributed to the decrease.

DHEA-S and A-dione are secreted from the adrenal glands and testes. No more than 10% of DHEA-S is of testicular origin [12]. Similarly, Kroboth et al. [19] showed that 5% of DHEA-S is secreted by the testes. Weinstein et al. [20] compared serum levels of sex steroids in peripheral veins and spermatic veins and found that the DHEA, A-dione, and T levels in the spermatic vein were 73.1, 30.7, and 751 ng/mL, respectively. These findings prove that DHEA and A-dione are secreted from the testes. Further, de Ronde et al. [21] measured adrenal androgen levels and estimated the percentage contributed by the testes. They stratified cases according to serum DHEA-S level and found that 0–14% of A-dione originated from the testes. In this study, DHEA-S decreased by about 24%, DHEA decreased by about 15%, and A-dione decreased by about 40%. These findings made us consider the role of the adrenal glands in the decrease in adrenal androgen levels after long-term GnRH antagonist treatment.

Several researchers have reported cases of ACTH-independent adrenal hyperplasia [12, 13], and the presence of functional LH receptors has been demonstrated in patients with ACTH-independent Cushing’s syndrome [13, 14]. These findings demonstrate that LH-RH agonist treatment reduces serum cortisol levels and reveal a relationship between the presence of LH receptors in the adrenal glands and cortisol production. LH receptors were identified on the reticular layer of adrenal cortex cells and demonstrated the presence of the cytochrome P450 side-chain cleavage enzyme in the same cells [22]. These results suggest that LH-positive adrenal cortex cells are steroidogenic. DHEA-S is produced in H295R adrenal cortical cells via functional LH receptors [23]. These findings suggest that LH affects the function of the adrenal glands and regulates the secretion of adrenal hormones.

Our group previously found a significant decrease in serum adrenal androgen levels in patients with a prostate carcinoma treated with a LH-RH agonist [16]. Using immunohistochemistry, we also discovered the presence of LH receptors on adrenal cortex cells in the reticular layer in patients treated with a LH-RH agonist. Furthermore, we found that the correlation between ACTH and DHEA-S levels shifted to an inverse relationship during the treatment period. These results show that reduced adrenal synthesis of androgens stimulates ACTH secretion through a feedback mechanism. Therefore, long-term GnRH antagonist treatment might reduce serum adrenal androgen levels via LH receptors.

We speculated the existence of another mechanism by which GnRH antagonists inhibit adrenal androgen production directly via GnRH receptor protein in the adrenal glands. In addition to its expression in the pituitary gland, GnRH receptor is expressed in lymphocytes and many extra-pituitary tissues, including breast, ovarian, and prostate [24]. Ziegler et al. [25] demonstrated that GnRH receptor is present in the adrenal glands at the mRNA and protein levels in normal human adrenal tissues, adrenocortical and adrenomedullary tumors, and adrenal cell lines. Although the presence of GnRH receptor in the adrenal glands suggests that adrenal androgen production was suppressed via GnRH receptor, it is unclear how the receptor works in the adrenal glands.

Bashin et al. [26] demonstrated that FSH levels began to rise towards pretreatment levels despite continued administration of a LH-RH agonist after achieving an initial nadir in young healthy men. They called this effect “FSH escape” but the mechanism is unknown. Santen et al. [27] reported the same increase in FSH after the administration of a LH-RH agonist in patients with prostate cancer. Crawford et al. [28] investigated the efficacy and safety of the GnRH antagonist degarelix compared to the LH-RH agonist leuprolide in the main trial CS21 and extension trial CS21A. The authors showed that the median FSH levels were 1.20 and 4.40 IU/L in the degarelix 240/80 mg and leuprolide groups, respectively (*p* < 0.0001) 1 year after initiating treatment. In this study, the serum LH and FSH levels reached a nadir 1 month after initiating treatment (LH: 0.285 ± 0.385, FSH: 0.889 ± 1.100; mean ± standard deviation). After reaching the nadir, the FSH levels began to rise gradually until 12 months after treatment started. We found a significant change between the FSH nadir and the level 12 months after initiating treatment. We observed very little “FSH escape”, but the FSH levels 12 months after initiating treatment were similar to data reported by Crawford et al. [28]. Continuous suppression of FSH caused by a GnRH antagonist has been discussed and its therapeutic advantage is under discussion [29]. Radu et al. [30] showed that FSH receptors are expressed by endothelial cells in a wide range of tumors, including prostate carcinomas. We are continuing to study the relationship between FSH and the prognosis of the patients examined in this study.

## Conclusions

In summary, we found a significant decrease in adrenal androgen levels in patients treated with a GnRH antagonist for 12 months. Considering the existence of functional LH receptors in cases of ACTH-independent Cushing’s syndrome or in human adrenal cortex cells, long-term GnRH antagonist administration may reduce serum adrenal androgen levels via LH receptors.

## Abbreviations

ACTH: Adrenocorticotropic hormone
A-dione: Androstenedione
DHEA: Dehydroepiandrosterone
DHT: Dihydrotestosterone
E_2_: 17β-estradiol
FSH: Follicle-stimulating hormone
GnRH: Gonadotropin-releasing hormone
LH: Luteinizing hormone
LH-RH: Luteinizing hormone-releasing hormone
T: Testosterone
